# Characterization of the *MMP9* Gene and Its Association with *Cryptocaryon irritans* Resistance Traits in *Trachinotus ovatus* (Linnaeus, 1758)

**DOI:** 10.3390/genes14020475

**Published:** 2023-02-13

**Authors:** Jun Liu, Ke-Cheng Zhu, Jin-Min Pan, Hua-Yang Guo, Bao-Suo Liu, Nan Zhang, Jing-Wen Yang, Dian-Chang Zhang

**Affiliations:** 1 Key Laboratory of South China Sea Fishery Resources Exploitation and Utilization, Ministry of Agriculture and Rural Affairs, South China Sea Fisheries Research Institute, Chinese Academy of Fishery Sciences, 231 Xingang Road West, Haizhu District, Guangzhou 510300, China; 2 Sanya Tropical Fisheries Research Institute, Sanya 572018, China; 3 Guangdong Provincial Engineer Technology Research Center of Marine Biological Seed Industry, Guangzhou 510300, China

**Keywords:** *Trachinotus ovatus*, *MMP9*, *Cryptocaryon irritans*, single nucleotide polymorphisms

## Abstract

The MMPs are endogenous proteolytic enzymes that require zinc and calcium as cofactors. *MMP9* is one of the most complex matrix metalloproteinases in the gelatinase family and has many biological functions. In mammals, mmp9 is thought to be closely associated with cancer. However, studies in fish have rarely been reported. In this study, to understand the expression pattern of the *ToMMP9* gene and its association with the resistance of *Trachinotus ovatus* to *Cryptocaryon irritans*, the sequence of the MMP9 gene was obtained from the genome database. The expression profiles were measured by qRT–PCR, the SNPs were screened by direct sequencing, and genotyping was performed. The *ToMMP9* gene contained a 2058 bp ORF encoding a putative amino acid sequence of 685 residues. The homology of the *ToMMP9* in teleosts was more than 85%, and the genome structure of *ToMMP9* was conserved in chordates. The *ToMMP9* gene was expressed in different tissues of healthy individuals and was highly expressed in the fin, the gill, the liver and the skin tissues. The *ToMMP9* expression in the skin of the infected site and its adjacent sites increased significantly after *C. irritans* infection. Two SNPs were identified in the *ToMMP9* gene, and the SNP (+400A/G) located in the first intron was found to be significantly associated with the susceptibility/resistance to *C. irritans*. These findings suggest that *ToMMP9* may play an important role in the immune response of *T. ovatus* against *C. irritans*.

## 1. Introduction

The MMPs are a class of endogenous proteolytic enzymes that require zinc ions and calcium ions as their cofactors and they are mainly synthesized and secreted by the neutrophils, the macrophages, the smooth muscle cells, the vascular endothelial cells and other cells [1]. They can hydrolyse type IV, type V, and type XI collagen and laminin, glycan core protein, etc., and are the only collagenases found thus far that can hydrolyse fibrous collagen [2]. The MMPs can degrade almost all of the ECM components and are the main degrading enzyme system of the ECM. Tumour invasion and metastasis, embryogenesis, ischaemia-hypoxia injury, atherosclerosis, the inflammatory response and other processes in the human body are all related to the MMPs [3]. There are currently approximately 26 MMPs, most of which exist in the human proteome [4,5]. Based on the conserved and specificity of the acting substrates, MMPs can be classified into six major groups [6,7]. They are collagenases, gelatinases, stromelysins, matrilysins, membrane type metalloproteinases and other MMPs. Collagenases mainly use collagen types I, II, III, VII and X as substrates and include MMP-1, MMP-8, MMP-13 and MMP-18. Gelatinases mainly use collagen type IV and gelatin as substrates and include MMP-2 and MMP-9. Both gelatinases are associated with a variety of pathologies [8,9]. Stromelysins mainly degrade laminin, fibronectin (FN), and proteoglycans, and their members include MMP-3, MMP-10, MMP-11, and MMP-17, and some of them are also involved in the activation of MMP zymogen form activation [10,11]. Matrilysins include MMP-7 and MMP-26, which play an important role in the degradation of ECM proteins such as type IV collagen, laminin, and nestin (Entactin), as well as in the processing of non-ECM proteins [10]. Membrane metalloproteinases (MT-MMPs), including MMP-14, MMP-15, MMP-16, MMP-24, MMP-17, and MMP-25, degrade type I, II, and IV collagen as well as FN [12]. Other MMPs, including MMP-12 (Macrophageelastase), MMP-19 (RASI-1), MMP-20 (Enamelysin), and MMP-23 (CA-MMP).

MMP9, discovered in 1974, is the most complicated matrix metalloproteinase in the gelatinase family [13]. It leads to gelatin and type IV, V, XI and XVI collagen degradation during tissue remodeling, which is essential for tumor invasion and metastasis [14]. Additional investigations have found that MMP9 serves an essential role in preventing apoptosis, degrading the ECM and promoting angiogenesis. MMP9 is secreted in its proenzyme form or inactive form from the endothelial cells, the leukocytes, the fibroblasts, the neutrophils and the macrophages. Synthesis of MMP9 generally occurs in the bone marrow during granulocyte differentiation [15]. It is activated in vitro by reactions with organomercurial agents and in vivo by a series of protease cascades. MMP9 has five structurally distinct structural domains, namely the hydrophobic signal peptide sequence, the N-terminal pre-peptide region, the catalytically active region, the proline-rich hinge region, and the C-terminal carboxyl region. The main role of the N-terminal prepeptide region is to maintain the stability of the zymogen. When this region is cut by exogenous enzymes, the MMPs zymogen is activated. There are zinc ion binding sites in the catalytically active region, which are essential for the catalytic action of the enzyme. While the C-terminal carboxyl region is related to the substrate specificity of the enzyme, where the enzyme catalytically active region and the pre-peptide region are highly conserved.

MMP9 has been studied more in mammals than in fish. Yoshinari et al. [16] revealed an increase in MMP9 expression in zebrafish at the injured edge of the epithelium following the early regeneration process. On this basis, LeBert et al. [17] demonstrated that the manifestation of MMP9 differently modulates the collagen reorganization in acute and chronic tissue lesions and recovery and in wound repair. The expression of MMP9 in *Red crucian carp*, *Danio rerio*, *Ctenopharyngodon idella* and *Pelteobagrus fulvidraco* was significantly increased after attack by *Aeromonas hydrophila* [18,19,20,21]. MMP9 is also involved in the antiparasitic response. The coinfection of salmon with *Lactobacillus salmon* and *Lepeophtheirus salmonis* showed that MMP9 was induced during the initial infection with Lactobacillus salmon. When the wound healing response changed from acute to chronic in severe infection and the louse reached an active stage, a second inducement of the antiparasitic response occurred [22,23]. Zhou et al. [24] found that the downregulation of miR-155-5p attenuated the brain injury induced by *Angiostrongylus cantonensis* infection and downregulated the MMP9 protein expression.

Single nucleotide polymorphism (SNP), refers to DNA sequence polymorphism caused by variation in a single nucleic acid at the genomic level. It is the most common type of heritable variation. In genomic DNA, any base can be mutated and SNPs are located in the coding or non-coding regions of genes and are genetic markers of disease susceptibility genes in the human genome, even susceptibility loci that can affect cancer, heart disease, diabetes and other common diseases. When SNPs are located in coding regions, when synonymous mutations occur, they do not change the protein sequence. However, due to the existence of codon preference in protein translation, from the common codon to the less common codon, the speed of ribosomal passage through the mRNA fragment around the SNP is changed, and the folding process in the cell is generally synchronized with translation, SNP affects protein folding and the time of its transfer to the cell membrane, thus changing the spatial structure and function of the protein [25]. When a missense mutation occurs it directly changes the composition of amino acids; when a nonsense mutation occurs, the codon becomes a stop codon and the peptide chain synthesis ends prematurely; a mutation in the stop codon causes the peptide chain to continue to lengthen, all of which lead to a change in the sequence of the protein, thus affecting its spatial structure and the stability of the protein and the functions it exercises. The non-coding region contains introns, promoters, enhancers, etc. SNPs in intron regions affect the splice site activity of mRNAs to influence gene function.

*T. ovatus* is a trevally with sweet-meat that has high nutritional value, growth speed and economic benefit. With the increase in the scale of domestic farming year by year, the coastal areas of South China have become the most important mariculture fishing areas [26]. The expansion and increased density as the breeding and aquaculture water body pollution degree deepens places farmed fish at risk of being infected with parasitic diseases and experiencing the harm of such infections [27]. The marine leukoplakia inflicted by *C. irritans* has emerged as a significant barrier to the reproduction of *T. ovatus*. To explore whether the *MMP9* gene serves as a defence in *C. irritans* infection, we identified the *MMP9* gene and examined the mRNA status of *MMP9* in healthy *T. ovatus*. We then analyzed the changes in *MMP9* expression after *C. irritans* infection, filtered out the relevant SNPs and evaluated the relevance between the SNPs and the tolerance toward *C. irritans*.

## 2. Materials and Methods

### 2.1. Infection Experiment and Sample Collection

The *C. irritans* were isolated from the *C. irritans*-infected T. ovatus at the Shenzhen experimental base of the South China Sea Fisheries Research Institute and reproduced with fish as hosts. At the beginning of the infection experiment, the prepared *T. ovatus* without parasite infection were put into 0.5m3 cylinders respectively as required, and the temperature and salinity of seawater in all the barrels were ensured to be the same. The infection test was carried out under the condition that the concentration of *C. irritans* reached 8000 theronts/L. At 48 hours after infection, skin tissues were obtained from 9 fish in the infection group and 9 fish in the control group. The control group was used prior to infection (PRE), and the infected group was divided into attached infected area skin (ATT) and adjacent infected skin (ADJ) groups. The samples were divided for two storage methods: direct freezing in liquid nitrogen and incubation at 4 °C overnight followed by storage at -80 °C with RNAlater. The samples were sent to Novogene (Guangzhou, CHN) for transcriptome sequencing (RNA-seq).

### 2.2. DNA/RNA Extraction and cDNA Synthesis

Fin DNA was collected with MagPure Tissue DNA DA Kit and the corresponding DNA extractor (Magen, Guangzhou, China). RNA was collected with HiPure Universal RNA Mini Kit (Magen, Guangzhou, China), and DNA synthesis with Evo M-MLV reverse transcription kit (Accurate Biology, Guangzhou, China). In addition, the concentration and mass of DNA and RNA were measured using a NanoDrop 2000 spectrophotometer (Thermo Fisher Scientific, Waltham, MA, USA) and a 1% agarose gel, separately.

### 2.3. Bioinformatics Analysis

The DNA and cDNA sequences of *T. ovatus MMP9* were obtained from the genome database [28]. Amino acid sequences were inferred from the Editseq module of the DNAstar software. Online tool SMART (http://SMART.embl-heidelberg.de/, accessed on 18 June 2022) was used to analyze the protein domains; Predict signal peptides using SignalP 5.0 (http://www.cbs.dtu.dk/services/SignalP/, accessed on 18 June 2022); GSDS 2.0 (http://GSDS.gao-lab.org/, accessed on 18 June2022) was used to predict gene structure; Multiple sequence homology comparisons between *T. ovatus* and other species were performed using DnaMan 9 software; and the maximum likelihood algorithm of Mega 7 software was used to construct a phylogenetic tree.

### 2.4. SNP Screening and Genotyping

Specific primers (Table 1) were designed to amplify *T. ovatus MMP9* genomic DNA sequences. Three resistant and three susceptible fish were selected for PCR amplification. The PCR products were purified and sequenced by Ruibiotech (Guangzhou, China). Based on the obtained sequences, possible SNP loci were inferred by sequence alignment and analysis. The primers (Table 1) were redesigned according to the selected SNP loci and amplified and sequenced in 50 resistant and 50 susceptible fish. To analyse the association between genotype frequency/allele frequency and *C. irritans* resistance traits, SPSS 22 software was used for the chi-square test, with *p* < 0.05 indicating statistically significant.

### 2.5. T. ovatus MMP9 Gene Expression Analysis

The tissue distribution of MMP9 mRNA in twelve healthy *T. ovatus* tissues was identified using quantitative real-time polymerase chain reaction (qRT–PCR). Primer sequences for *T. ovatus MMP9* and the control gene *EF-1α* are listed in Table 1. The qRT–PCR procedure consisted of one step of 95 °C for 5 min; 40 cycles of 95 °C for 10 s, 58 °C for 45 s, 95 °C for 5 s, 56 °C for 60 s, and 95 °C for 1 s; and a final step of 50 °C for 8 min. The 2^−ΔΔCT^ method was used to evaluate the relative expression [29]. In SPSS 22.0 software, one-way ANOVA was used to analyse the data by Duncan’s test. Data are presented as the mean ± SE of three replicates, with differences considered significant at *p* < 0.05 and extremely significant at *p* < 0.01.

The relative expression of *T. ovatus MMP9* in skin after infection with *C. irritans* was evaluated using the fragments per kilobase of exon model per million mapped fragments (FPKM) value. FeatureCounts was used to calculate reads mapped to each gene and the FPKM value for each gene was based on the length of the gene and reads mapped to that gene. The differential expression of PRE vs. ATT, PRE vs. ADJ, and ATT vs. ADJ was analysed by DESeq2 software. The screening criteria for differentially expressed genes were |log2(FoldChange)| > 1 & padj <= 0.05. The Benjamini–Hochberg method was used to adjust the resulting *p* value to control for the false discovery rate.

## 3. Results

### 3.1. ToMMP9 Sequence Characteristics

**Figure 1:**
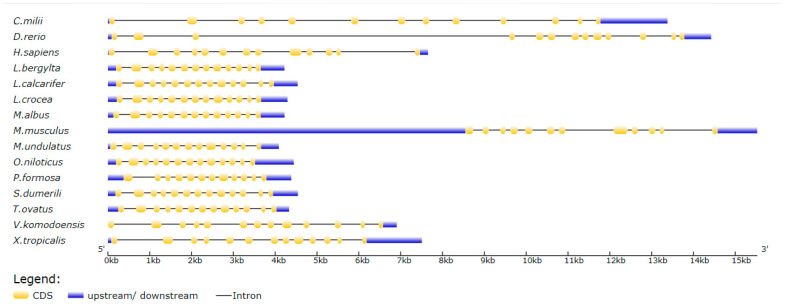
MMP9 Gene structure of Callorhinchus milii, Danio rerio, Homo sapiens, Labrus bergylta, Larimichthys crocea, Lates calcarifer, Melopsittacus undulatus, Monopterus albus, Mus musculus, Oreochromis niloticus, Poecilia formosa, Seriola dumerili, Trachinotus ovatus, Varanus komodoensis, Xenopus tropicalis.

**Figure 2:**
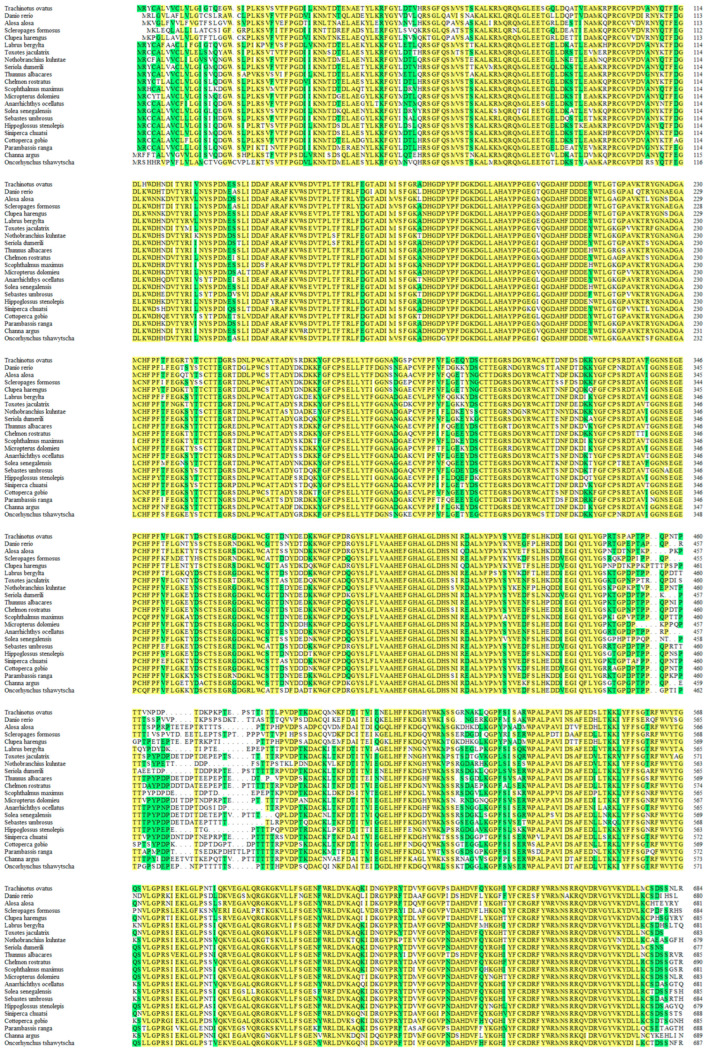
Multiple alignment of the deduced amino acid sequences of MMP9 in *T. ovatus* and other bony fishes. The homology level is highlighted, with yellow indicating more than 50% homology and green indicating more than 33% homology.

The genome sequence of *ToMMP9* was 4330 bp and contained 13 exons and 12 introns (Figure 1). The open reading frame was 2058 bp in length and encoded a 685-amino acid peptide. The predicted molecular weight of *ToMMP9* was 77.17 kDa, and the theoretical isoelectric point was 5.424. The signal peptide sequence of the *ToMMP9* protein was 1–21 aa, and it was followed by three consecutive fibronectin type 2 domains (FN2, 225–273 aa; 283–331 aa; and 341–389 aa) and four consecutive haemopexin-like repeat domains (HX, 497–541 aa; 543–584 aa; 589–635 aa; and 637–677 aa). The sequence similarity of ToMMP9 in the teleosts was approximately 85% (Figure 2). *ToMMP9* had the highest homology with *Seriola dumerili MMP9* (87.1%) and *Thunnus albacares MMP9* (85.2%) (Figure 2).

### 3.2. ToMMP9 Phylogeny and Structural Analysis

**Figure 3:**
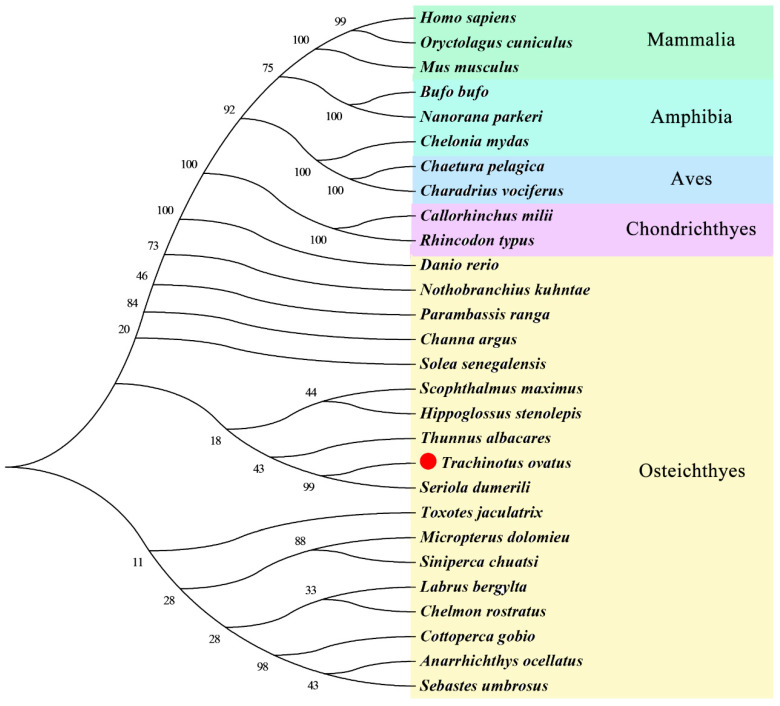
Phylogenetic analysis of *MMP9* from selected teleost species.

The phylogenetic analysis was performed using the *MMP9* amino acid sequence (Figure 3) to reveal homology among different species. The results demonstrated that *ToMMP9* was closely related to *S. dumeriliMMP9* and clustered with *T. albacares MMP9* and formed a clade with other bony fishes, in agreement with the usual taxonomy among all species examined.

The genomic structure of MMP9 in chordates is shown in Figure 1. According to the results, a high level of genomic structure of MMP9 was conserved among species. Most of the chordate MMP9 genomic sequences contained 12 introns and 13 exons, with the exception of *Mus musculus* and *Poecilia formasa*, which each had 12 exons and 11 exons.

### 3.3. ToMMP9 Expression Profile in Healthy Tissues and Expression Pattern after C. irritans Infection

**Figure 4:**
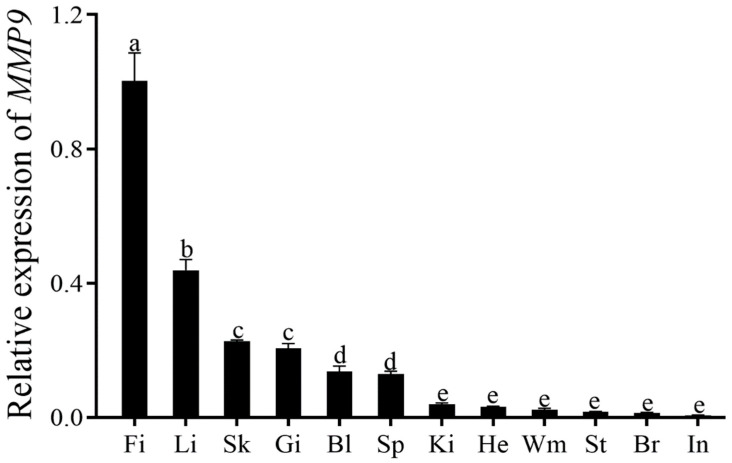
Tissue distributions of *ToMMP9* in blood (Bl), brain (Br), fin (Fi), gill (Gi), head-kidney (Ki), intestine (In), liver (Li), skin (Sk), spleen (Sp), stomach (St) and white muscle (Wm). Different letters indicate significant differences.

The relative expression of mRNA in 12 organs was measured by qRT–PCR (Figure 4) to confirm the role of *ToMMP9*. The *ToMMP9* expression level was the highest in the fin and the liver, followed by the skin and the gill, and was lower in the stomach, the brain and the intestine.

**Figure 5:**
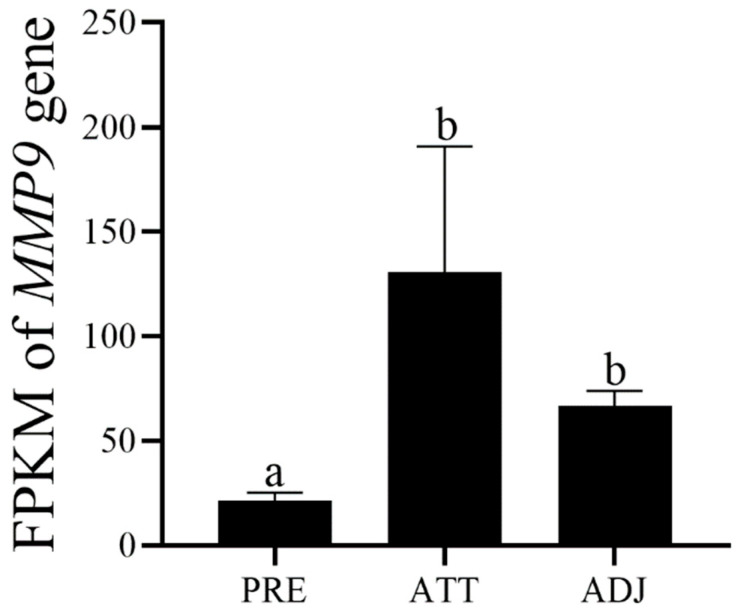
The expression of *ToMMP9* in skin after *C. irritans* infection. PRE means prior to infection, ATT means attached infected area skin and ADJ means adjacent infected skin. Different letters indicate significant differences.

To analyse the possible role of *ToMMP9* in the defence against *C. irritans* infection, we calculated the FPKM values of ToMMP9 in the three groups (ATT, ADJ, and PRE) following transcriptome sequencing and analysed the significance of the differences among them (Figure 5).

### 3.4. Correlation Analysis of ToMMP9 SNPs and Resistance Characteristics of C. irritans

In total, two SNPs were identified and validated in *ToMMP9*, (−1340A/C) and (+400A/G); SNP (−1340A/C) was in the promoter, and SNP (+400A/G) was located in the first intron (Table 2). Chi-square tests revealed significant differences in the genotype frequencies and the allele frequencies of the SNPs (+400A/G) among both susceptible and tolerant groups (Table 3). However, there was no significant difference in the SNP (−1340A/C).

## 4. Discussion

In this study, the *ToMMP9* gene was identified and characterized. The length of the *ToMMP9* gene was 4330 bp, and the ORF length was 2058, encoding 685 amino acids. The MMP9 protein contained a conserved Pro-Arg-Cys-Gly-Val/ASN-PRO-ASP (PRCGV/NDP) sequence immediately following the signal peptide (aa 1–21), and the conserved cysteine residue in that sequence has been found to play a crucial role in the enzymatic activation of most MMPs [30]. The MMP protein also contained three nearly identical fibronectin repeats (FN2), whose function is to promote the degradation of large or gelatinous substrates, such as elastin and gelatine [31]. In addition, the haemagglutinin domain of the ToMMP9 protein shared only 25–33% sequence similarity with the haemagglutinin domain of other members of the matrix metalloproteinase family [32], illustrating its special position in the family. The clustering of the *ToMMP9* gene sequence with the *MMP9* genes of other bony fishes (Figure 3) and homologous amino acid sequence alignment results (Figure 2) showed that *ToMMP9* shares more than 85% similarity with other homologous genes.

The tissue expression profiles of the *MMP9* gene have been more focused on mammalian tissues rather than on fish tissues. The mRNA expression level of *MMP9* was elevated in the appendix, the bone marrow and the lymph nodes of the *Homo sapiens*; and in the thymus, the spleen, and the lung of *M. musculus* and *Rattus norvegicus* [33,34,35]. Yoong et al. [36] found in zebrafish that *MMP9* is widely expressed in circulating white blood cells that are different from the macrophages from 24 h after fertilization, and that these white blood cells migrate gradually to the site of injury. In adult zebrafish, the most predominant expression of *MMP9* is in the splenic region. In addition, *MMP9* is highly expressed in the blood, the kidney and the skin of *Oreochromis niloticus* [37]. Thus, MMP9 is highly expressed in the immune-related tissues in both mammals and fish, suggesting that the *MMP9* gene may be closely related to the immune function. In this study, a high expression of *ToMMP9* was observed in the fin, the liver, the skin and the gill tissues of *T. ovatus*. The skin, the gills and the fins are the main tissues of the fish that *C. irritans* parasitizes [38]. The larva of *C. irritans* invades and damages the surface of the body, and the trophozoites feed on the tissue fluid and the cells of the fish [39]. Findings from this study demonstrated it is likely that *MMP9* has a major role in the innate immunity of *T. ovatus*. A key detoxification organ of aquatic animals is the liver. Wang et al. [40] found that the liver of *Paralichthys olivaceus* infected with *C. irritans* will appear with obvious congestion phenomenon. Superoxide dismutase (SOD) and Catalase (CAT) are important enzymes for scavenging reactive oxygen species (ROS), which can reduce the oxidation and phagocytosis of damaged cells [41,42]. Yi et al. [43] found that the activities of SOD and CAT in the liver of the *Sebastiscus marmoratus* infected with the *C. irritans* increased significantly from 24 to 96 h after infection. As such, it is inferred that the increase of SOD and CAT activities in the liver may play an active role in controlling the damage of the host cells caused by the ROS. The high expression of *ToMMP9* in the liver may be related to the immune function of the liver.

*C. irritans* can cause leukoplakia in mariculture fish with a high mortality rate, which is a big obstacle to the evolution of *T. ovatus* farming. [38,44]. Zhu et al. [45] identified an upregulation of two IFN signaling pathway genes (IRF1 and IRF2) in the skin and the gills of *T. ovatus* after infection with *C. irritans.* In addition, Li et al. [46] concluded that *C. irritans* infection can induce an increase in the number of antigen-presenting cells and lymphocytes in the mucosal immune system, which can mediate the activation of local specific B cells, leading to antibody production. Wang et al. [47] found that the serum and skin mucus of *Siganus oramin* exhibited a potent lethal effect on both the larvae and the trophozoites of *C. irritans*, suggesting a role for mucosal immunity in *C. irritans* infections. The expression of *ToMMP9* was dramatically increased in the skin of the infected/adjacent sites after infection with *C. irritans*, which suggested that *MMP9* might be involved in the response of fish to *C. irritans* resistance through the innate and the mucosal immunity. However, the mechanism by which *ToMMP9* functions and its specific regulatory mechanism are not clear.

The SNP variants located within genes have the potential to effect protein structure or expression levels explicitly, and thus could reflect some determinants of disease or trait-related genetic mechanisms [48]. In this study, two SNPs in the *MMP9* gene were detected and verified. Significant distinctions in the genotype and allele fraction frequencies of the SNPs located in the first intron (+400A/G) were found among the susceptible and resistant groups. At present, increasing attention has been given to the role of introns in genes. Wei et al. [49] screened 17 SNPs derived from the first intron of *EGFR*, which is a growth-related gene in *Sinonovacula constricta*. Guo et al. [50] investigated the association of *ToLAAO/ToLAAO-like* genes with *C. irritans* resistance traits and identified five SNPs, and two SNPs in LAAO (6200C/T and 6237G/A) were notably linked to resistance traits in *C. irritans*. Wang et al. [51] identified four SNPs in the *β_2_m* gene of *Megalobrama amblycephala*, and the allele and genotype frequencies of SNP (+408A/C) located on the intron were significantly associated with antibacterial traits. Zhu et al. [52] examined and validated 11 SNPs in four RAC genes of *T. ovatus*, of which six SNPs resided within introns. The SNP (+6864T/G) in the fifth intron of *Torac1a* as a major correlate of resistance traits in *C. irritans* was found. In addition, some studies have proven that introns can play a role by regulating downstream genes [53,54].

## 5. Conclusions

In summary, the *ToMMP9* genes were characterized, and their associations with *T. ovatus* resistance traits were analysed in this study. *ToMMP9* is commonly expressed in twelve organs and is highly expressed in the fins and the liver. *ToMMP9* expression in the skin of the infected site and the adjacent sites increased significantly after *C. irritans* infection. In addition, two SNPs were identified in the *ToMMP9* gene, and the SNP (+400A/G) located in the first intron was found to be significantly associated with susceptibility/resistance in *C. irritans*. However, further studies are needed to reveal the regulatory mechanism of *ToMMP9* in the fight against *C. irritans*.

## Figures and Tables

**Table 1:** Primers used in this study.

Primer	Sequence (5′–3′)
PMMP9-F	ATGGCTGCGGCTCTT
PMMP9-R	TCCTGCGTGCCTATCC
MMP9-F	GAGCATTCCCCTCAAGTCCG
MMP9-R	GCCGCAGATTCCCAGGTTTA
S-PMMP9-F	TCCATTGCAGCCGCTTGTA
S-PMMP9-R	TGCATGTTGCCTAATCCATAATCT
S-MMP9-F	GGCAGAAGTGAGTAGCA
S-MMP9-R	TGACACCATAGACTGGAAGC
qPCR-MMP9-F	CACCAGTGAGGGACGAG
qPCR-MMP9-R	GGCTGCCACCAGAAACA
EF-1α F	CCCCTTGGTCGTTTTGCC
EF-1α R	GCCTTGGTTGTCTTTCCGCTA

**Table 2:** The SNP sites of *MMP9* and *Lamc2* genes in *T. ovatus*.

Gene	Locus	Tybe	Changes of DNA Base
MMP9	promoter	transversion	A/C
	intron	transition	A/G

**Table 3:** Distribution of *T. ovatus MMP9* SNPs in the resistant and susceptible groups.

Gene	Position	Genotype	Susceptible	Resistant	X^2^ (*p*)	Allele	Susceptible	Resistant	X^2^ (*p*)
MMP-9	A/C	AA	17	21	0.68 (0.71)	A	58	63	0.52 (0.47)
		CC	9	8		C	42	37	
		AC	24	21					
	A/G	AA	14	3	9.07 (0.01)	A	50	31	7.49 (0.006)
		GG	14	22		G	50	69	
		AG	22	25					

Note: The statistically significant differences and highly significant differences are denoted with *p* <0.05 and *p* <0.01, respectively.

## Data Availability

No data was used for the research described in the article.

## References

[1] Maral S., Acar M., Balcik O.S., Uctepe E., Hatipoglu O.F., Akdeniz D., Altun H.U., Kosar A., Gunduz M., Gunduz E. (2015). Matrix Metalloproteinases 2 and 9 Polymorphism in Patients with Myeloproliferative Diseases: A STROBE-Compliant Observational Study. Medicine.

[2] Qorri B., Kalaydina R.V., Velickovic A., Kaplya Y., Decarlo A., Szewczuk M.R. (2018). Agonist-Biased Signaling via Matrix Metalloproteinase-9 Promotes Extracellular Matrix Remodeling. Cells.

[3] Rath T., Roderfeld M., Graf J., Roeb E. (2009). Matrix metalloproteinases in inflammatory bowel disease-from basic research to clinical significance. Z. Gastroenterol..

[4] Amin S.A., Adhikari N., Jha T. (2017). Is dual inhibition of metalloenzymes HDAC-8 and MMP-2 a potential pharmacological target to combat hematological malignancies?. Pharmacol. Res..

[5] Fields G.B. (2015). New strategies for targeting matrix metalloproteinases. Matrix Biol..

[6] Fanjul-Fernandez M., Folgueras A.R., Cabrera S. (2010). Matrix metalloproteinases: Evolution, gene regulation and functional analysis in mouse models. Biochim. Biophys. Acta..

[7] Kapoor C., Vaidya S., Wadhwan V. (2016). Seesaw of matrix metalloproteinases (MMPs). J. Cancer Res. Ther..

[8] Notomi T., Okayama H., Masubuchi H. (2000). Loop-mediated isothermal amplification of DNA. Nucl. Acids Res..

[9] Egeblad M., Werb Z. (2002). New functions for the matrix metalloproteinases in cancer progression. Nature Reviews Cancer.

[10] Barksby H.E., Milner J.M., Patterson A.M. (2006). Matrix metalloproteinase 10 promotion of collagenolysis via procollagenase activation: Implications for cartilage degradation in arthritis. Arthritis Rheum..

[11] Geurts N., Martens E., Van Aelst I. (2008). β-hematin interaction with the hemopexin domain of gelatinase B/MMP-9 provokes autocatalytic processing of the propeptide, thereby priming activation by MMP-3. Biochemistry.

[12] Velasco G., Cal S., Merlos-Suarez A. (2000). Human MT6-matrix metalloproteinase: Identification, progelatinase A activation, and expression in brain tumors. Cancer Res..

[13] Bronisz E., Kurkowska-Jastrzębska I. (2016). Matrix Metalloproteinase 9 in Epilepsy: The Role of Neuroinflammation in Seizure Development. Mediators Inflamm..

[14] Liu Y., Liu H., Luo X., Deng J., Pan Y., Liang H. (2015). Overexpression of SMYD3 and matrix metalloproteinase-9 are associated with poor prognosis of patients with gastric cancer. Tumour. Biol..

[15] Yabluchanskiy A., Ma Y., Iyer R.P., Hall M.E., Lindsey M.L. (2013). Matrix metalloproteinase-9: Many shades of function in cardiovascular disease. Physiology.

[16] Yoshinari N., Ishida T., Kudo A., Kawakami A. (2009). Gene expression and functional analysis of zebrafish larval fin fold regeneration. Dev. Biol..

[17] LeBert D.C., Squirrell J.M., Rindy J., Broadbridge E., Lui Y., Zakrzewska A., Eliceiri K.W., Meijer A.H., Huttenlocher A. (2015). Matrix metalloproteinase 9 modulates collagen matrices and wound repair. Development.

[18] Xiong N.X., Luo S.W., Fan L.F., Mao Z.W., Luo K.K., Liu S.J., Wu C., Hu F.Z., Wang S., Wen M. (2021). Comparative analysis of erythrocyte hemolysis, plasma parameters and metabolic features in red crucian carp (*Carassius auratus red var*) and triploid hybrid fish following Aeromonas hydrophila challenge. Fish Shellfish Immunol..

[19] Liyanage T.D., Nikapitiya C., Lee J., De Z.M. (2020). Potential immune regulatory role of miR-146a upon *Aeromonas hydrophila* and *Edwardsiella piscicida* infections in zebrafish. Braz. J. Microbiol..

[20] Xu X.Y., Shen Y.B., Fu J.J., Liu F., Guo S.Z., Li J.L. (2013). Characterization of MMP-9 gene from grass carp (*Ctenopharyngodon idella*): An Aeromonas hydrophila-inducible factor in grass carp immune system. Fish Shellfish Immunol..

[21] Ke F., Wang Y., Hong J., Xu C., Chen H., Zhou S.B. (2015). Characterization of MMP-9 gene from a normalized cDNA library of kidney tissue of yellow catfish (*Pelteobagrus fulvidraco*). Fish Shellfish Immunol..

[22] Fast M.D. (2014). Fish immune responses to parasitic copepod (namely sea lice) infection. Dev. Comp. Immunol..

[23] Skugor S., Glover K.A., Nilsen F., Krasnov A. (2008). Local and systemic gene expression responses of Atlantic salmon (*Salmo salar L.*) to infection with the salmon louse (*Lepeophtheirus salmonis*). BMC Genom..

[24] Zhou X., Zhang J., Liu J., Guo J., Wei Y., Li J., He P., Lan T., Peng L., Li H. (2021). MicroRNA miR-155-5p knockdown attenuates Angiostrongylus cantonensis-induced eosinophilic meningitis by downregulating MMP9 and TSLP proteins. Int. J. Parasitol..

[25] Komar A.A. (2007). Genetics: SNPs, silent but not invisible. Science.

[26] Sun L.Y., Guo H.Y., Zhu C.Y., Ma Z.H., Jiang S.G., Zhang D.C. (2014). Genetic polymorphism of breeding populations of golden pompano (*Trachinotus ovatus*). South China Fish..

[27] Dan X.M., Li A.X., Lin X.T., Bo J.S., Zhang H.F. (2008). Immune response and immunoprotection of pompanos (*Trachinotus ovatus*) against *Cryptocaryon irritans*. Acta Hydrobiol. Sin..

[28] Zhang D.C., Guo L., Guo H.Y., Zhu K.C., Li S.Q., Zhang Y., Zhang N., Liu B.S., Jiang S.G., Li J.T. (2019). Chromosome-level genome assembly of golden pompano (*Trachinotus ovatus*) in the family Carangidae. Sci Data.

[29] Livak K.J., Schmittgen T.D. (2001). Analysis of relative gene expression data using real-time quantitative PCR and the 2(-Delta Delta C(T)) Method. Methods.

[30] Matrisian L.M. (1990). Metalloproteinases and their inhibitors in matrix remodeling. Trends Genet..

[31] Vandooren J., Geurts N., Martens E., VandenSteen P.E., Jonghe S.D., Herdewijn P., Opdenakker G. (2011). Gelatin degradation assay reveals MMP-9 inhibitors and function of O-glycosylated domain. World J. Biol. Chem..

[32] Dufour A., Sampson N.S., Li J., Kuscu C., Rizzo R.C., Deleon J.L., Zhi J., Jaber N., Liu E., Zucker S. (2011). Small-molecule anticancer compounds selectively target the hemopexin domain of matrix metalloproteinase-9. Cancer Res..

[33] Fagerberg L., Hallström B.M., Oksvold P., Kampf C., Djureinovic D., Odeberg J., Habuka M., Tahmasebpoor S., Danielsson A., Edlund K. (2014). Analysis of the human tissue-specific expression by genome-wide integration of transcriptomics and antibody-based proteomics. Mol. Cell Proteom..

[34] Yue F., Cheng Y., Breschi A., Vierstra J., Wu W., Ryba T., Sandstrom R., Ma Z., Davis C., Pope B.D. (2014). A comparative encyclopedia of DNA elements in the mouse genome. Nature.

[35] Yu Y., Fuscoe J.C., Zhao C., Guo C., Jia M., Qing T., Bannon D.I., Lancashire L., Bao W., Du T. (2014). A rat RNA-Seq transcriptomic BodyMap across 11 organs and 4 developmental stages. Nat. Commun..

[36] Yoong S., O’Connell B., Soanes A., Crowhurst M.O., Lieschke G.J., Ward A.C. (2007). Characterization of the zebrafish matrix metalloproteinase 9 gene and its developmental expression pattern. Gene Expr. Patterns.

[37] Brawand D., Wagner C.E., Li Y.I., Malinsky M., Keller I., Fan S., Simakov O., Ng A.Y., Lim Z.W., Bezault E. (2014). The genomic substrate for adaptive radiation in African cichlid fish. Nature.

[38] Colorni A., Burgess P. (1997). *Cryptocaryon irritans* Brown 1951, the cause of ‘white spot disease’ in marine fish: An update. Aquar. Sci. Conserv..

[39] Diggles B.K., Lester R.J.G. (1996). Infections of *Cryptocaryon irritans* wild fish from southeast Queensland, Australia. Dis. Aquat. Org..

[40] Wang Y.G., Liu Z.W., Lin C.Y., Cheng X., Wang L., Li H. (2011). Cryptocaryoniosis in cultured turbot and its treatment. J. Fish. China.

[41] Zeinali F., Homaei A., Kamrani E. (2015). Sources of marine superoxide dismutases: Characterist and applications. Int. J. Biol. Macromol..

[42] Hadwan M.H. (2018). Simple spectrophotometric assay for measuring catalase activity in biologi tissues. BMC Biochem..

[43] Yin F., Gong Q., Li Y., Dan X., Sun P., Gao Q., Shi Z., Peng S., Li A. (2014). Effects of Cryptocaryon irritans infection on the survival, feeding, respiratory rate and ionic regulation of the marbled rockfish Sebastiscusmarmoratus. Parasitology.

[44] Dan X.M., Li A.X., Lin X.T., Teng N., Zhu X.Q. (2006). A standardized method to propagate *Cryptocaryon irritans* on a susceptible host pompano *Trachinotus ovatus*. Aquaculture.

[45] Zhu K.C., Zhang N., Liu B.S., Guo L., Guo H.Y., Jiang S.G., Zhang D.C. (2020). Functional Analysis of IRF1 Reveals its Role in the Activation of the Type I IFN Pathway in Golden Pompano, *Trachinotus ovatus* (Linnaeus 1758). Int. J. Mol. Sci..

[46] Li Y.W., Jiang B., Dan X.M., Li A.X. (2019). Advances in the research on mucosal immune response offish against *Cryptocaryon irritans* infection. J. Fish. China.

[47] Wang F.H., Xie M.Q., Li A.X. (2010). A novel protein isolated from the serum of rabbitfish (*Siganus oramin*) is lethal to *Cryptocaryon irritans*. Fish Shellfish Immunol..

[48] Collins F.S., Guyer M.S., Charkravarti A. (1997). Variations on a theme: Cataloging human DNA sequence variation. Science.

[49] Wei K.Y., Xie S.M., Wang S.T., Chen Y.K., Niu D.H., Li J.L. (2019). Polymorphism of SNPs in *EGFR* intron 1 and its association with growth traits in Sinonovacula constricta. J. Fish. China.

[50] Guo L., He P.Y., Zhu K.C., Guo H.Y., Liu B.S., Zhang N., Jiang S.G., Zhang D.C. (2022). Functional identification of *ToLAAO* genes and polymorphism association analysis of *Cryptocaryon irritans* resistance in *Trachinotus ovatus*. Aquac. Res..

[51] Wang J.X., Luo H., Sun Q.H., Wang H.L., Liu H. (2021). Characterization of *β2m* gene and its association with antibacterial trait in *Megalobrama amblycephala*. Aquaculture.

[52] Zhu K.C., Liu J., Liu B.S., Guo H.Y., Zhang N., Guo L., Jiang S.G., Zhang D.C. (2022). Functional characterization of four ToRac genes and their association with anti-parasite traits in *Trachinotus ovatus* (Linnaeus, 1758). Aquaculture.

[53] Morgan J.T., Fink G.R., Bartel D.P. (2019). Excised linear introns regulate growth in yeast. Nature.

[54] Parenteau J., Maignon L., Berthoumieux M., Catala M., Gagnon V., Abou Elela S. (2019). Introns are mediators of cell response to starvation. Nature.

